# Hypertension outcomes in a fragile setting: predictors of blood pressure reduction and control in the Central African Republic

**DOI:** 10.3389/fpubh.2025.1664189

**Published:** 2025-10-29

**Authors:** Anna Maria Doro Altan, Boris Tchenebou, Kevine Iffio, Gabriella Bortolot, Stefano Orlando, Giovanni Guidotti, Sandro Petrolati, Pierre Somse, Fausto Ciccacci

**Affiliations:** ^1^Link Campus University, Rome, Italy; ^2^Community of Sant'Egidio, DREAM Program, Bangui, Central African Republic; ^3^Community of Sant'Egidio, DREAM Program, Rome, Italy; ^4^Department of Biomedicine and Prevention, University of Rome Tor Vergata, Rome, Italy; ^5^ASL Roma 1, Rome, Italy; ^6^Department of Cardioscience, San Camillo Hospital, Rome, Italy; ^7^Minister of Health, Bangui, Central African Republic

**Keywords:** hypertension, Africa, diabetes, primary care, implementation

## Abstract

**Background:**

Hypertension is a leading contributor to cardiovascular disease and early mortality, and its impact is growing rapidly in low-income countries. In the Central African Republic, the condition represents a major and under-addressed health problem. This study examines the outcomes of hypertension care in Bangui, focusing on factors influencing blood pressure control and reduction (CAR).

**Methods:**

This cross-sectional study analyzed clinical records of hypertensive patients receiving care in Bangui. Demographic and clinical data were collected at baseline and at the most recent follow-up (October-November 2024) to identify factors associated with BP control (<140/90 mmHg) and reduction (decrease of ≥20 mmHg in systolic or ≥10 mmHg in diastolic BP).

**Results:**

We included 656 patients (69% female, median age 59 years). BP control and clinically significant BP reduction were achieved in 39.5 and 86.7% of patients. Diabetes was an independent predictor of lower BP control (OR = 0.36; 95%CI:0.25–0.52; *p* < 0.001) and lower BP reduction (OR = 0.56; 95%CI:0.35–0.88; *p* = 0.012). Chronic kidney disease was associated with lower BP reduction (OR = 0.10; 95%CI:0.02–0.52; *p* = 0.006). Higher baseline hypertension correlated with BP reduction (Grade 3 hypertension: OR = 88.3; 95%CI:23.4–587; *p* < 0.001). Older age was associated with BP reduction (OR = 1.16; 95%CI:1.04–1.29; *p* = 0.007).

**Conclusion:**

In Bangui, structured hypertension care proved feasible and led to significant BP reductions, although target control rates remained low, particularly in patients with diabetes and CKD. Strengthening follow-up and access to tailored treatment could improve outcomes in this fragile setting.

## Background

High blood pressure is among the most important modifiable determinants of cardiovascular disease, stroke, and early death, contributing to an estimated 10.8 million deaths worldwide in 2019 (1). Although prevalence has plateaued in wealthier nations, low- and middle-income countries now carry the majority of cases—around three quarters of the global burden (2). In sub-Saharan Africa, hypertension prevalence ranges widely between 15 and 70%, with an average rate of 30% (3). This variability reflects the region's diverse socioeconomic and healthcare landscapes, highlighting significant challenges for health systems to implement effective and sustainable control measures.

In Africa, the challenge of hypertension is projected to intensify, driven by urban growth, lifestyle transitions, and fragile health systems. Estimates suggest that the number of affected individuals will grow from 130 million in 2010 to 216 million by 2030 (4). Prevalence is consistently higher in cities than in rural areas, reflecting differences in diet, activity levels, and psychosocial stress (5). Addressing these disparities requires tailored approaches that account for the unique characteristics of each setting. The health consequences of hypertension in sub-Saharan Africa are profound. For example, in Tanzania, hypertension-related conditions account for 33.9% of deaths from non-communicable diseases (6).

The Central African Republic (CAR), ranked among the least developed nations globally, carries a heavy burden of hypertension compounded by poverty, fragile health services, and persistent political instability. Data from older adults (≥65 years) show a prevalence of 61.1%, with less than half aware of their diagnosis, under one in five on treatment, and fewer than a quarter achieving control (7). Similar trends are observed in neighboring countries, such as Uganda, where rural populations often experience higher rates of undiagnosed hypertension due to restricted healthcare access (8).

The WHO considers hypertension a central priority in its strategy to curb noncommunicable diseases (NCDs), promoting the HEARTS technical package to reinforce primary care in resource-constrained environments (9). Yet, evidence on how structured hypertension programs function in fragile or post-conflict settings, such as CAR, remains scarce.

This study aimed to evaluate the effectiveness of hypertension management in Bangui, CAR. Specifically, the study focused on identifying factors associated with blood pressure (BP) control and clinically significant reductions in BP among patients, offering a model for scaling up chronic disease management in similar settings across sub-Saharan Africa.

## Methods

This study employed a retrospective longitudinal design, with repeated measures at two points in time (baseline and most recent visit).

The study was conducted at the DREAM center in Bangui, CAR. The center is part of the DREAM program, which provides comprehensive chronic disease management in resource-limited settings (10–12). The program's integration of community-based care with advanced diagnostics and treatment offers valuable insights into addressing the dual challenges of accessibility and effectiveness in chronic disease management. In July 2019, the DREAM Program expanded its outpatient center to include a dedicated hypertension service within its outpatient center in Bangui.

The intervention aligns with the WHO HEARTS technical package, incorporating lifestyle counseling, protocol-driven treatment, guaranteed access to essential medicines, team-based care, and digital tools for monitoring and follow-up (9). In the CAR few public health facilities provide systematic care for hypertension or chronic diseases.

Clinical data are collected during routine follow-up visits at the DREAM center. For the purpose of the study, for each patient, demographic and clinical variables were collected at two time points: at the start of their care (baseline) and at their most recent visit in October-November 2024. Given the retrospective nature of the study, only variables systematically available in clinical records were included in the models. These comprise demographic factors, BMI, comorbidities (diabetes, CKD), hypertension grade, and treatment duration, which represent recognized predictors of hypertension outcomes in similar contexts

We included all medical records of individuals receiving hypertension care at the center in November 2024. Data were then retrospectively retrieved from their initial enrollment in care, which varied across patients, starting from July 2019 when the hypertension service was first established. All included patients had both baseline and follow-up BP measurements available, so no records were excluded for missing data. This approach allowed us to assess changes over time by comparing variables from the baseline visit with those from the most recent follow-up. Given the retrospective nature of the study, follow-up intervals varied due to clinical and logistical factors. To ensure consistency, only the first and most recent BP measurements were included, avoiding biases associated with selecting arbitrary time points. Fixed intervals were not used, as they would have led to missing data and inconsistent follow-up durations. This approach allowed each patient to contribute comparable data while capturing meaningful BP trends. The time between the two measurements was also considered in the analysis to account for differences in treatment duration.

Patients were included if they were adults aged 18 years or older, had a confirmed diagnosis of hypertension, and were actively receiving treatment at the DREAM center in November 2024. Exclusion criteria included incomplete medical records or missing baseline BP measurements, or pregnancy at the time of enrolment, given the specific impact of pregnancy on blood pressure. The study included all eligible hypertensive patients through a census approach, covering the entire target population under care at the center.

Blood pressure was measured at each visit by trained health personnel using validated automated devices, following international and national recommendations. Patients were seated, with the arm supported at heart level. Three measurements were taken at 1–2 min intervals, and the average was recorded in the clinical chart.

BP measurements were extracted from patients' first and most recent visits to evaluate two key outcomes: BP control and BP clinical reduction. BP control was defined as achieving BP values < 140/90 mmHg according to international guidelines (13). Clinically significant BP reduction was defined as a reduction of ≥20 mmHg in systolic BP or ≥10 mmHg in diastolic BP. This threshold was selected based on evidence from clinical research and international guidelines, which have demonstrated that such reductions are associated with a meaningful decrease in cardiovascular risk and events, including stroke and myocardial infarction (13–16).

Additional data extracted included demographic characteristics (age, sex), clinical variables (BMI, diabetes status, chronic kidney disease [CKD] status), and treatment duration. BMI was categorized into four classes (underweight, normal weight, overweight, obese), and blood pressure grades were classified using established hypertension guidelines (grade 1: systolic blood pressure 140–159 mmHg and/or diastolic 90–99 mmHg; grade 2: systolic 160–179 mmHg and/or diastolic 100–109 mmHg; grade 3: systolic ≥180 mmHg and/or diastolic ≥110 mmHg) (13).

### Statistical analysis

Descriptive statistics summarized demographic and clinical characteristics. Continuous variables (age, BMI, treatment duration) were reported as medians with interquartile ranges (IQRs). Categorical variables (sex, diabetes status, CKD status, BMI classes, hypertension grades) were presented as counts and percentages. Group comparisons were performed using: The Mann-Whitney U test for continuous variables, the chi-squared test for categorical variables. Separate multivariate logistic regression models were developed to identify factors associated with the two primary outcomes. Independent variables included age, sex, BMI classes, diabetes status, CKD status, hypertension grades, and treatment duration. Results were reported as odds ratios (ORs) with 95% confidence intervals (CIs). Statistical significance was set at *p* < 0.05. To better evaluate the association of age with outcomes, analyses included both continuous age and age grouped in 5-year increments. Additionally, results were stratified by BMI classes and hypertension grades to explore subgroup differences. All analyses were conducted using R Studio Version 2024.09.1+394.

This study adhered to the ethical principles outlined in the Declaration of Helsinki. As anonymized routine clinical data were used, the study was exempted from formal ethical approval. Patient confidentiality was strictly maintained throughout the study.

## Results

The analyzed cohort consisted of 656 hypertensive patients (*n* = 656) at their first visit, among whom 69% were female (*n* = 454) and 31% male (*n* = 202).

The characteristics of the cohort, along with univariate analyses for both outcome variables, are presented in Table 1, while Table 2 reports the results of the multivariate logistic regression models.

**Table 1:** Cohort characteristics and univariate analysis for blood pressure control (BP < 140/90) and clinically significant blood pressure reduction (systolic BP reduction 20 mmHg, or diastolic BP reduction 10 mmHg).

	**Blood Pressure Control (BP**<**140/90)**				**Blood Pressure Clinical Reduction (SBP reduction 20 mmHg, or DBP reduction 10 mmHg)**			
**Characteristic**	***N*** =656^*^	**YES** = **259**+	**NO** = **397**+	***p*** **-value**	***N*** = 656^*^	**YES** = **569**+	**NO** = **87**+	***p*** **-value**
Sex (*n*, %)				0.462				1.000
F	454 (69%)	184 (71%)	270 (68%)		454 (69%)	394 (69%)	60 (69%)	
M	202 (31%)	75 (29%)	127 (32%)		202 (31%)	175 (31%)	27 (31%)	
BMI Classes (*n*, %)				0.159				0.022
Underweight	36 (5.5%)	23 (8.9%)	13 (3.3%)		36 (5.5%)	32 (5.6%)	4 (4.6%)	
Normal weight	253 (39%)	101 (39%)	152 (38%)		253 (39%)	226 (40%)	27 (31%)	
Overweight	204 (31%)	80 (31%)	124 (31%)		204 (31%)	177 (31%)	27 (31%)	
Obese	163 (25%)	55 (21%)	108 (27%)		163 (25%)	134 (24%)	29 (33%)	
Hypertension grade (*n*, %)								
High-Normal	0 (0%)	0 (0%)	0 (0%)		0 (0%)	0 (0%)	0 (0%)	
Hypertension Grade 1	261 (40%)	107 (41%)	154 (39%)		261 (40%)	191 (34%)	70 (80%)	
Hypertension Grade 2	224 (34%)	81 (31%)	143 (36%)		224 (34%)	209 (37%)	15 (17%)	
Hypertension Grade 3	171 (26%)	71 (27%)	100 (25%)		171 (26%)	169 (30%)	2 (2.3%)	
Age (median, IQR)	59 (51–66)	60 (53–66)	59 (51–66)	**0.047**	59 (51–66)	60 (52–66)	57 (49–66)	0.329
BMI (median, IQR)	25.8 (22.4– 29.9)	25.2 (21.6–29.2)	26.1 (22.9–30.6)	**0.000**	25.8 (22.4–29.9)	25.7 (22.4–29.6)	27.0 (22.7–31.2)	**0.000**
Time in treatment, months (median, IQR)	31 (16–50)	32 (16–51)	30 (15–49)	**0.010**	31 (16–50)	31 (16–50)	31 (15–48)	0.210
Diabetes (*n*, %)				0.462				0.097
N	442 (67%)	206 (80%)	236 (59%)		442 (67%)	402 (71%)	40 (46%)	
Y	214 (33%)	53 (20%)	161 (41%)		214 (33%)	167 (29%)	47 (54%)	
Chronic kidney disease (*n*, %)				**0.004**				0.098
N	647 (99%)	258 (100%)	389 (98%)		647 (99%)	564 (99%)	83 (95%)	
Y	9 (1.4%)	1 (0.4%)	8 (2.0%)		9 (1.4%)	5 (0.9%)	4 (4.6%)	

^*^n (%).

+n (%); Median (Q1–Q3). Bold values indicate statistically significant results at the 5% level (*p* < 0.05).

**Table 2:** Multivariate analysis of factors associated with blood pressure control (BP < 140/90) and clinically significant blood pressure reduction (systolic BP reduction 20 mmHg, or diastolic BP reduction 10 mmHg).

	**Multivariate for Blood Pressure Control (BP**<**140/90)**			**Blood Pressure Clinical Reduction (SBP reduction 20 mmHg, or DBP reduction 10 mmHg)**		
**Characteristic**	**OR** ^1^	**95% CI** ^1^	***p*** **-value**	**OR** ^1^	**95% CI** ^1^	***p*** **-value**
Age_5	1.05	0.98, 1.13	0.2	1.16	1.04, 1.29	0.007
**Diabete**						
N	–	–		–	–	
Y	0.36	0.25, 0.52	**< 0.001**	0.56	0.35, 0.88	0.012
**CKD**						
N	–	–		–	–	
Y	0.22	0.01, 1.21	0.2	0.10	0.02, 0.52	0.006
**BP_class**						
High-Normal	–	–		–	–	
Hypertension Grade 1	1.09	0.57, 2.14	0.8	2.71	1.42, 5.21	0.003
Hypertension Grade 2	0.76	0.39, 1.51	0.4	13.8	6.33, 31.2	< 0.001
Hypertension Grade 3	0.88	0.45, 1.78	0.7	88.3	23.4, 587	< 0.001

^1^OR, Odds Ratio; CI, Confidence Interval. Bold values indicate statistically significant results at the 5% level (*p* < 0.05).

The distribution of BMI at start of assistance indicated that 39% were normal weight (*n* = 253), 31% overweight (*n* = 204), 25% obese (*n* = 163), and 5.5% underweight (*n* = 36). Most patients (40%, *n* = 261) were initially diagnosed with Grade 1 hypertension, followed by 34% with Grade 2 (*n* = 224) and 26% with Grade 3 (*n* = 171). Furthermore, 33% of the cohort had diabetes (*n* = 214), and 1.4% had CKD (*n* = 9). The median age of the patients was 59 years (IQR: 51–66), and the median duration of treatment was 31 months (IQR: 16–50) (Table 1).

### Blood pressure control (BP < 140/90 mmHg)

Blood pressure control, defined as achieving values below 140/90 mmHg, was observed in 39.5% of the cohort (*n* = 259). In univariate analysis (Table 1), the presence of CKD was associated with a lower likelihood of BP control (*p* = 0.004). Additionally, patients who achieved BP control had a slightly longer treatment duration (median: 32 vs. 30 months, *p* = 0.010), and a weak association was observed with age (*p* = 0.047). Diabetes was not significantly associated with BP control in univariate analysis (*p* = 0.462), nor were sex (*p* = 0.462) or BMI categories (*p* = 0.159).

In the multivariate analysis (Table 2), diabetes emerged as an independent predictor of lower BP control (OR = 0.36; 95% CI: 0.25–0.52; *p* < 0.001). The association with CKD was no longer significant after adjustment (OR = 0.22; 95% CI: 0.01–1.21; *p* = 0.2). Age and treatment duration were not retained as significant in the adjusted model. No other variables, including sex and BMI, were independently associated with BP control.

### Clinically significant blood pressure reduction (SBP ≥ 20 mmHg or DBP ≥ 10 mmHg)

A clinically significant blood pressure reduction was achieved in 86.7% of patients (*n* = 569). In descriptive analysis as reported in Table 1, patients with Grade 3 hypertension had the highest proportion of BP reduction (*n* = 169/171, 98.8%), followed by Grade 2 (*n* = 209/224, 93%) and Grade 1 (*n* = 191/261, 73,1%). Obese patients had a slightly higher proportion of BP reduction (*p* = 0.022), while CKD patients had a lower proportion (*p* = 0.098). No significant associations were found for sex (*p* = 1.000) or diabetes (*p* = 0.097).

In the multivariate analysis (Table 2), diabetes (OR = 0.56; 95% CI: 0.35–0.88; *p* = 0.012) and CKD (OR = 0.10; 95% CI: 0.02–0.52; *p* = 0.006) were independently associated with a lower likelihood of achieving a clinically significant BP reduction. Given the very small size of the CKD subgroup (*n* = 9), the corresponding odds ratios should be interpreted with caution.

Higher hypertension grade was strongly associated with greater BP reduction, with patients with Grade 2 hypertension having an OR of 13.8 (95% CI: 6.33–31.2; *p* < 0.001) and those with Grade 3 hypertension having an OR of 88.3 (95% CI: 23.4–587; *p* < 0.001) compared to those with high-normal BP. Older age was also independently associated with a higher likelihood of BP reduction (OR = 1.16; 95% CI: 1.04–1.29; *p* = 0.007). Obesity, while showing a trend in descriptive analysis, was not retained as an independent predictor in the multivariate model.

**Figure 1:**
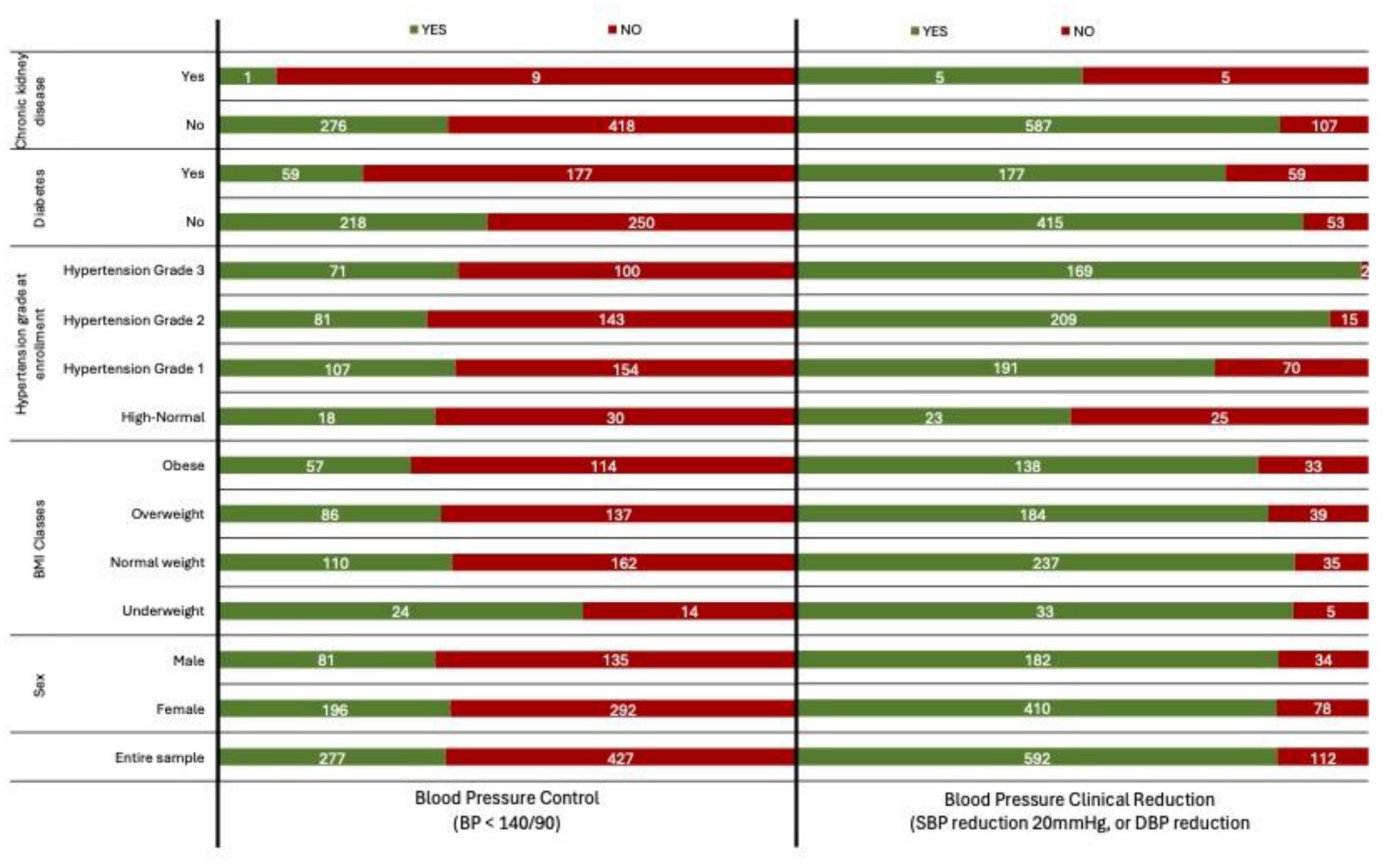
Distribution of patients by demographic and clinical characteristics, stratified by blood pressure control (BP < 140/90) and clinically significant blood pressure reduction (systolic BP reduction 20 mmHg, or diastolic BP reduction 10 mmHg).

Figure 1 illustrates the distribution of patients by demographic and clinical characteristics stratified by outcomes, highlighting the relationships between comorbidities and treatment success.

## Discussion

Our findings suggest that structured hypertension care can be successfully implemented in fragile settings such as Bangui, CAR, though replication in other low-income or post-conflict contexts requires further investigation. Among the hypertensive patients included in the analysis, a significant portion achieved measurable improvements in BP outcomes. Notably, while 86.7% of the patients achieved a clinically significant BP reduction, only 39.5% reached the target BP level. This indicates that while substantial reductions in BP were common, achieving clinical targets remained a challenge. The chronic and often asymptomatic course of hypertension could likely contribute to the observed gap between BP reduction and BP control. Patients may show marked improvements from very high baseline levels without reaching target values, partly due to reduced adherence, clinical inertia, and irregular follow-up. In fragile health systems, these challenges could be compounded by cultural factors, as health literacy is often focused more on infectious diseases than on chronic conditions, limiting awareness and long-term engagement in hypertension care.

Our finding aligns with previous studies on hypertension in sub-Saharan Africa, where blood pressure control is generally below 10% and there is considerable variability across different regions (2).

The finding that patients with Grade 3 hypertension achieved the strongest BP reductions but still had relatively modest control rates is consistent with the fact that, starting from very high baseline values, large absolute decreases are more likely, yet many remain above target thresholds. While our analysis relied on standard hypertension grades in accordance with international guidelines, further stratification was not feasible given the limited sample size in some subgroups. Patients with severe hypertension may require longer follow-up and more intensive management to achieve control.

Previous studies have highlighted that achieving therapeutic targets for hypertension in Africa is hindered not only by limited healthcare services but also by persistent barriers such as unreliable drug supply and limited patient education on cardiovascular risk (17). While major public health investments have focused on infectious diseases, non-communicable diseases have received less attention, contributing to low control rates across the region. Contextual factors such as conflict-related stress, food insecurity, and the growing availability of salt-rich processed foods may further exacerbate these challenges, although they could not be directly assessed in our study.

Some community-based interventions have been implemented in African countries, demonstrating significant benefits in terms of blood pressure reduction, increased healthcare service uptake, and improved screening and diagnosis rates (18). Recent studies have shown that community-based strategies, such as the involvement of local healthcare workers and integrated care models, can significantly improve treatment adherence and blood pressure control (2). However, the available evidence remains limited, and the interventions implemented thus far have been highly heterogeneous.

Our analysis showed that diabetes was an independent predictor of both lower BP control and reduced likelihood of achieving a clinically significant reduction, consistent with findings from cohorts in Uganda, and Tanzania (19). Other studies across sub-Saharan Africa similarly reported that the coexistence of diabetes complicates hypertension care and worsens prognosis (20). Similarly, CKD was strongly associated with poorer outcomes, in line with evidence linking renal impairment to reduced BP response and higher cardiovascular risk in African populations (21). These findings highlight the need to tailor hypertension care to high-risk patients, particularly those with diabetes or CKD, within broader chronic disease management programs.

Moreover, more severe hypertension grades at baseline were associated with larger reductions, likely reflecting greater therapeutic margins; a similar pattern was confirmed in large-scale meta-analyses of BP-lowering interventions (14).

Finally, we observed a modest but significant association between older age and BP reduction, which may relate to adherence patterns or more consistent follow-up in older patients, as reported in community-based programs in Ethiopia and other African settings (18). Altogether, our findings align with the broader literature while adding evidence from a fragile, post-conflict context where such data remain scarce.

The effectiveness of antihypertensive therapy in our patients may also be influenced by therapeutic choices. Previous studies have highlighted that clinical inertia and difficulties in accessing medications represent key barriers to hypertension control in African settings (22). Moreover, the widespread use of monotherapy in many regions of sub-Saharan Africa, despite recommendations for combination therapies, could explain the low control rates observed in the continent (23). However the data about the therapeutic choices in our sample were not available.

This study has several limitations. First, its retrospective design, based on routine clinical records, limited the availability of variables such as drug type, dosage, treatment changes, adherence, and key laboratory parameters (lipids, HbA1c, renal function), restricting the assessment of overall cardiovascular risk. Moreover, this limit may have influenced the internal consistency of outcome estimates across patients and limits comparability with other similar studies. Second, the independent variables were restricted to those systematically recorded (age, sex, BMI, diabetes, CKD, hypertension grade, treatment duration), while potentially relevant factors such as lifestyle or socioeconomic status were missing, reducing the depth of analysis. Third, although the study had repeated measures, follow-up intervals varied across patients, limiting the evaluation of temporal dynamics. More advanced approaches (e.g., survival or mixed-effects models) were not feasible given the limited number of time points and missing intermediate data. In addition, while multivariable regressions were applied, no formal diagnostic tests (e.g., multicollinearity, goodness-of-fit, outlier analysis) were performed; such checks would be important in larger studies. Fourth, comorbidities like diabetes and CKD were identified from clinical charts rather than systematic screening as we did elsewhere (24), likely underestimating their prevalence; moreover, the very small CKD subgroup (*n* = 9) makes related estimates unstable. In addition, limited diagnostic capacity (e.g., restricted availability of laboratory testing for diabetes or CKD and use of non-standardized BP measurement devices) may have contributed to underreporting or misclassification of comorbidities. However, the fact that significant improvements were observed despite these constraints reinforces the real-world validity of the findings.

Fifth, the absence of a comparison group prevents direct assessment of program effectiveness. Sixth, loss to follow-up may have excluded patients with poorer outcomes; by design we included only patients still in care in November 2024, which may introduce a survivor bias, as those who died or discontinued treatment were not captured. Finally, the study was conducted in a single urban center in Bangui, limiting generalizability to rural areas or other fragile settings.

Despite these constraints, the study offers rare real-world evidence on hypertension management in a post-conflict African country.

## Conclusion

This study confirms that structured hypertension care is feasible and can yield substantial clinical results even in extremely fragile health systems. The use of standard antihypertensives, electronic medical records, community engagement, and referral agreements within a chronic care framework contributed to high rates of clinically meaningful blood pressure reduction. Although BP control remains more challenging—especially among patients with diabetes and CKD—this experience highlights the potential for replicating simplified, protocol-based hypertension management within existing health service structures. Further research is essential to evaluate the long-term effectiveness of these interventions and to develop tailored strategies suited to the specific needs of sub-Saharan Africa.

## Data Availability

The raw data supporting the conclusions of this article will be made available by the authors, without undue reservation.
